# TGF-β1 increases cellular invasion of colorectal neuroendocrine carcinoma cell line through partial epithelial-mesenchymal transition

**DOI:** 10.1016/j.bbrep.2022.101239

**Published:** 2022-03-01

**Authors:** Norihiko Sasaki, Seiichi Shinji, Yuuki Shichi, Toshiyuki Ishiwata, Tomio Arai, Takeshi Yamada, Goro Takahashi, Ryo Ohta, Hiromichi Sonoda, Akihisa Matsuda, Takuma Iwai, Kohki Takeda, Kazuhide Yonaga, Koji Ueda, Sho Kuriyama, Toshimitsu Miyasaka, Hiroshi Yoshida

**Affiliations:** a Research Team for Geriatric Medicine (Vascular Medicine), Tokyo Metropolitan Institute of Gerontology, Tokyo, 173-0015, Japan; b Department of Gastrointestinal and Hepato-Biliary-Pancreatic Surgery, Nippon Medical School, Tokyo, 113-8603, Japan; c Division of Aging and Carcinogenesis, Research Team for Geriatric Pathology, Tokyo Metropolitan Institute of Gerontology, Tokyo, 173-0015, Japan; d Department of Pathology, Tokyo Metropolitan Geriatric Hospital and Institute of Gerontology, Tokyo, 173-0015, Japan

**Keywords:** Neuroendocrine carcinoma, EMT, TGF-β1, α2-integrin, Adhesion, Invasion, BSA, bovine serum albumin, CSC, cancer stem cell, EMT, epithelial-to-mesenchymal transition, FACS, fluorescence activated cell sorter, MFI, mean fluorescence intensity, MMP, matrix metalloproteinase, NEC, neuroendocrine carcinoma, NENs, neuroendocrine neoplasms, qRT-PCR, quantitative reverse transcription-polymerase chain reaction, SD, standard deviation, SEM, scanning electron microscopic, TGF, transforming growth factor-beta

## Abstract

Epithelial–mesenchymal transition (EMT) plays a pivotal role in cancer progression and metastasis in many types of malignancies, including colorectal cancer. Although the importance of EMT is also considered in colorectal neuroendocrine carcinoma (NEC), its regulatory mechanisms have not been elucidated. We recently established a human colorectal NEC cell line, SS-2. In this study, we aimed to clarify whether these cells were sensitive to transforming growth factor beta 1 (TGF-β1) and whether EMT could be induced through TGF-β1/Smad signaling, with the corresponding NEC cell-specific changes in invasiveness. In SS-2 cells, activation of TGF-β1 signaling, as indicated by phosphorylation of Smad2/3, was dose-dependent, demonstrating that SS-2 cells were responsive to TGF-β1. Analysis of EMT markers showed that mRNA levels changed with TGF-β1 treatment and that E-cadherin, an EMT marker, was expressed in cell-cell junctions even after TGF-β1 treatment. Invasion assays showed that TGF-β1-treated SS-2 cells invaded more rapidly than non-treated cells, and these cells demonstrated increased metalloproteinase activity and cell adhesion. Among integrins involved in cell-to-matrix adhesion, α2-integrin was exclusively upregulated in TGF-β1-treated SS-2 cells, but not in other colon cancer cell lines, and adhesion and invasion were inhibited by an anti-α2-integrin blocking antibody. Our findings suggest that α2-integrin may represent a novel therapeutic target for the metastasis of colorectal NEC cells.

## Highlights

• NEC cell line SS-2 is responsive to TGF-β1.
• TGF-β1 stimulation induces partial EMT, maintaining E-cadherin in SS-2 cells.
• TGF-β1-treated SS-2 cells exhibit increase in metalloproteinase activity and cell adhesion.
• α2-integrin is exclusively upregulated in TGF-β1-treated SS-2 cells.
• Use of anti-α2-integrin blocking antibody inhibits enhanced adhesion and invasion.

## 1 Introduction

Neuroendocrine neoplasms (NENs) are rare tumors originating from neuroendocrine cells that are widely distributed in the human body [1,2]. In 2017, an International Agency for Research on Cancer and World Health Organization experts consensus proposal defined three types of neuroendocrine tumors, two types of neuroendocrine carcinoma (NEC), and mixed neuroendocrine-non-neuroendocrine neoplasm [3]. According to the epidemiological database established by the Surveillance, Epidemiology, and End Results study in the United States, the incidence of NEN increased 6.4 times between 1973 and 2012, as did the number of locally localized cases [4]. Clinically, NEC is the most aggressive type of malignancy among NENs. NEC shows not only high proliferative capacities with a median Ki-67 labeling index of 80%, but also high metastatic capacity [5]. Novel therapeutic options are urgently required for patients with NEC because of NEC's resistance to chemotherapy and the increasing global incidence [6].

Epithelial-to-mesenchymal transition (EMT) is involved in cancer progression [7], and describes the process through which epithelial cells acquire a mesenchymal phenotype. Many studies have demonstrated that EMT plays a pivotal role in cancer progression and metastasis in many types of malignancies, including colorectal cancer (CRC) [8]. The process of EMT requires the cooperation of a complex network of factors, including molecular signaling pathways and regulators [9]. The regulation of EMT in CRC has been well studied. Upregulation of EMT-related signaling, such as transforming growth factor (TGF)-β1 and EMT transcription factors, downregulates epithelial cell junction proteins, including E-cadherin, and upregulates mesenchymal proteins, including N-cadherin, leading to a mesenchymal phenotype [10]. In addition, several small non-coding RNAs or microRNAs have been shown to be involved in the regulation of EMT in colorectal cancer (CRC) [10]. However, the regulatory mechanisms of EMT in highly metastatic colon NEC have not yet been elucidated.

Colorectal malignancies with solid or alveolar structures are rare, including poorly differentiated adenocarcinomas, medullary carcinomas, and NEC [11,12]. However, the histopathological and clinical characteristics of colorectal NEC and NEC from other sources have not been fully investigated because of the paucity of clinical data and established cell lines. NEC cell lines from the ascending colon are very rare, and include HROC57 established by another group and SS-2 from our group [6,13]. Experimental evidence has shown that the expression of cancer stem cell (CSC) markers is positively correlated with drug resistance [14]. SS-2 cells are more resistant to anti-CRC drugs than conventional CRC cells [6]. When SS-2 cells were seeded into ultra-low attachment plates, they formed spheres that expressed higher levels of the CSC marker CD133 compared to SS-2 cells cultured under adherent conditions [6], indicating that CD133 may play a role in drug resistance in SS-2 cells.

As NEC cell lines have recently been established, the features of SS-2 cells have not been fully elucidated. To date, there have been no reports on the role of EMT in colorectal NEC. In this study, we aimed to clarify whether TGF-β1 induced EMT in SS-2 cells and whether EMT mediated by TGF-β1/Smad signaling contributed to the invasiveness of the cells.

## 2 Materials and methods

### 2.1 Cell culture

The human NEC cell line SS-2 was previously established [6]. The SS-2 cells and other conventional CRC cell lines, HT-29-Luc (JCRB Cell Bank, Osaka, Japan), DLD-1 (Cell Resource Center for Biomedical Research, Tohoku University), Colo-320 (Riken BRC Cell Bank), and Caco-2 (Riken BRC Cell Bank), were grown in RPMI 1640 medium containing 10% fetal bovine serum (Thermo Fisher Scientific, Waltham, MA, USA) at 37 °C in a humidified 5% CO_2_ atmosphere. For EMT induction, the cells were cultured in growth medium with 10 ng/mL TGF-β1 (Peprotech, Rocky Hill, NJ, USA) for 48 h.

### 2.2 Immunoblotting

Immunoblotting was performed as described previously [15,16]. The following primary antibodies were used: monoclonal rabbit anti-pSmad2 (#3108; Cell Signaling Technology, Danvers, MA, USA), monoclonal rabbit anti-pSmad3 (ab52903; Abcam, Cambridge, UK), monoclonal rabbit anti-Smad2/3 (#8685; Cell Signaling Technology), polyclonal anti-TGFβR-I (SAB4502958; Sigma-Aldrich, St. Louis, MO, USA), polyclonal anti-TGFβR-II (sc-220; Santa Cruz Biotechnology, Dallas, TX, USA), monoclonal mouse anti-α2-integrin (sc-74466; Santa Cruz Biotechnology), and monoclonal mouse anti-β-actin (A5316; Sigma-Aldrich). All immunoblotting experiments were performed three times.

### 2.3 Scanning electron microscopic (SEM) analyses

Cells were fixed with 2.5% glutaraldehyde in 0.1 M phosphate buffer for 30 min at 4 °C, followed by 1% OsO_4_ dissolved in distilled water for 30 min at 4 °C. After dehydration using a graded ethanol series, the cells were coated with a platinum layer using an MSP-1S sputter coater (Shinku Device, Ibaraki, Japan), and photographed using a Phenom Pro desktop scanning electron microscope (Thermo Fisher Scientific).

### 2.4 Quantitative reverse transcription-polymerase chain reaction (qRT-PCR)

qRT-PCR analysis was performed as described previously [[15], [16], [17]]. Total RNA was isolated from cells using a RNeasy Plus Mini Kit (QIAGEN, Hilden, Germany) and was subsequently reverse-transcribed using the ReverTra Ace® qPCR RT Kit (Toyobo, Osaka, Japan). qRT-PCR was performed using the Power Sybr® Green kit (Applied Biosystems, Foster City, CA, USA) and the StepOnePlus™ real-time PCR system (Applied Biosystems). The primer sets used for qRT-PCR are listed in Table 1.

**Table 1:** Primer sets used for qRT-PCR.

Gene	Forward primer	Reverse primer
*MMP2*	GCGGCGGTCACAGCTACTT	TTCAGACTTTGGTTCTCCAGCTT
*MT1-MMP*	GAAGGATGGCAAATTCGTCTTC	AGGGACGCCTCATCAAACAC
*β1-integrin*	TTGGATTCTCCAGAAGGTGGTT	TCAGTGATCCACAAACTGCAACT
*α1-integrin*	TGCTCTCAATCAGACAAGGTTTG	GAGATGAACAGCACGTCTGCTT
*α2-integrin*	TGCCCCGAGCACATCAT	CGCAAATCCAAAGAGTTGACAA
*α6-integrin*	ACAGAAAGTGTGCATGGAGGAAA	TGGGAATGGGACGCAGTT
*E-cadherin*	CCAGTGAACAACGATGGCATT	TGCTGCTTGGCCTCAAAAT
*N-cadherin*	TGGGAATCCGACGAATGG	GCAGATCGGACCGGATACTG
*Snail*	CCCCAATCGGAAGCCTAACT	GCTGGAAGGTAAACTCTGGATTAGA
*Slug*	TGCGGCAAGGCGTTTT	TCTCCCCCGTGTGAGTTCTAA
*Vimentin*	TCCAAACTTTTCCTCCCTGAAC	GGGTATCAACCAGAGGGAGTGA
*β-actin*	GGTCATCACCATTGGCAATGAG	TACAGGTCTTTGCGGATGTCC

MMP: matrix metalloproteinase; qRT-PCR: quantitative reverse transcription-polymerase chain reaction.

### 2.5 Immunostaining

The cells were fixed with 4% (w/v) paraformaldehyde and then washed. Subsequently, the cells were blocked with phosphate buffered saline containing 1% (w/v) bovine serum albumin (BSA) and 5% (v/v) normal goat serum. After washing, the cells were incubated with polyclonal anti-E-cadherin antibody (Bioss, China) at 4 °C overnight. After washing, the cells were stained with Alexa Fluor® 488-conjugated secondary antibodies (Molecular Probes) and counterstained with 4′,6-diamidino-2-phenylindole. Immunofluorescence images were acquired using a confocal laser scanning microscope (Leica Microsystems, Wetzlar, Germany).

### 2.6 Invasion assay

Invasion assays were performed as described previously [[15], [16], [17]]. Cell culture inserts (pore size: 12 μm; diameter: 12 mm; Merck Millipore, Ltd., County Cork, Ireland) were coated with 80 μl of 2% Matrigel (Becton Dickinson, Franklin Lakes, NJ, USA) and used for the assay. Cells were plated at a density of 1.5 × 10^5^ cells/500 μL on the upper surface of the inserts and incubated for 24 h. Cells that had migrated through the membrane to the lower surface of the filter were fixed and stained with a Diff-Quick staining kit (Polysciences, Inc., Warrington, PA, USA), and then imaged using Mantra, multi-spectral microscopy. The images were loaded into the inForm software ver. 2.4 (Perkin-Elmer, Inc., Waltham, MA, USA) to count the number of invaded cells in 12 random fields with a 20x magnification objective.

### 2.7 Matrix metalloproteinase (MMP) gelatin zymography

MMP gelatin zymography analysis was performed as previously [17]. The same amounts of media samples were resolved on 8% SDS-PAGE gels containing 4.0 mg/mL gelatin. The gels were rinsed with a wash buffer (50 mM Tris-HCl, pH 7.5, 5 mM CaCl_2_, 1 μM ZnCl_2_, 2.5% Triton X-100) and soaked in an incubation buffer (50 mM Tris-HCl, pH 7.5, 5 mM CaCl_2_, 1 μM ZnCl_2_, 1% Triton X-100) at 37 °C for 24 h. After incubation, the gels were fixed and stained with Coomassie R-250, washed, and scanned.

### 2.8 Fluorescence activated cell sorter (FACS) analysis

FACS analysis was performed as described previously [17]. We used the following fluorescein isothiocyanate (FITC)-conjugated antibodies and primary antibodies: FITC-conjugated isotype control (Becton Dickinson), FITC-conjugated anti-α2-integrin (BioLegend), and anti-β1-integrin (Abcam). Mean fluorescence intensity (MFI) was calculated by subtracting the intensities of the controls.

### 2.9 Adhesion assay

Adhesion assay was performed as previously [17]. A total of 5 × 10^4^ cells/well were added to plates, and they were incubated for 1 h. After washing the plates with phosphate buffered saline, bound cell numbers were determined through ATP assays performed using a CellTiter-Glo® 2.0 Assay kit (Promega, Madison, WI, USA). The percentage of adhesive cells was calculated as the percentage of luminescence values in adherent cells relative to that in total cells.

### 2.10 Statistical analysis

Quantitative data are presented as the mean ± standard deviation (SD). Statistical analyses were performed using EZR (Saitama Medical Centre, Jichi Medical University; http://www.jichi.ac.jp/saitama-sct/SaitamaHP.files/statmedEN.html; Kanda, 2012). Unpaired Student's *t*-test and one-way ANOVA with Tukey's HSD test were used to compare two or more groups, respectively. Differences were considered statistically significant at *p* < 0.05.

## 3 Results and discussion

### 3.1 TGF-β1 signaling in NEC cell line SS-2

**Fig. 1:**
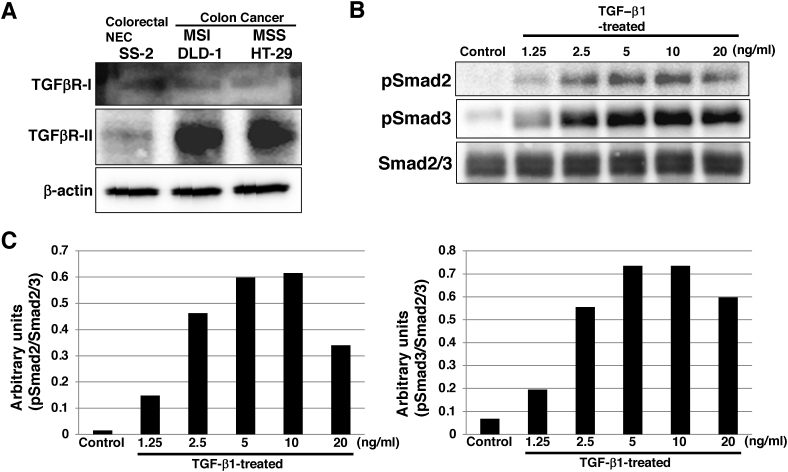
**TGF-β1 signaling in NEC cell line SS-2.** (A) Immunoblotting for TGFβR-I, TGFβR-II and β-actin (loading control) in SS-2, DLD-1, and HT-29 cells. (B) Immunoblotting for p-Smad2, p-Smad3 and Smad2/3 in SS-2 cells cultured with or without indicated concentration of TGF-β1 for 2 d. (C) The histogram shows mean densitometric readings for the proteins normalized to Smad2/3. NEC: neuroendocrine carcinoma; TGF- β: transforming growth factor-beta.

It has not been investigated whether NEC cells are responsive to TGF-β1. Using western blot analysis, we first examined the expression of TGF-β1 receptors and found that both TGFβR-I and TGFβR-II were expressed in SS-2 cells, but the expression level of TGFβR-II was weak compared with that in colon cancer cell lines DLD-1 and HT-29 (Fig. 1A). We then examined the responsiveness of SS-2 cells to TGF-β1. Activation of TGF-β1 signaling, as indicated by the phosphorylation of Smad2 and 3, was observed in a dose-dependent manner up to 10 and 5 ng/mL, respectively (Fig. 1B and C). These results demonstrated that the NEC cell line SS-2 was responsive to TGF-β1.

### 3.2 EMT of NEC cell line SS-2

**Fig. 2:**
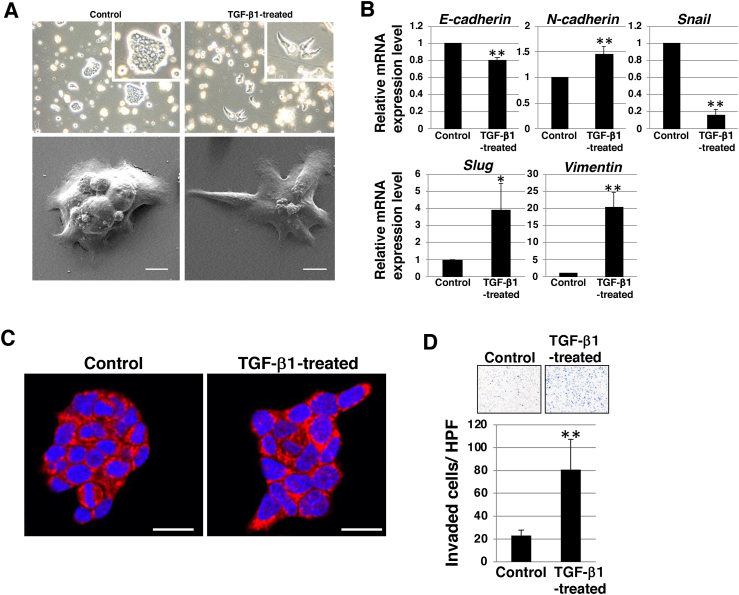
**EMT of NEC cell line SS-2.** (A) Representative phase contrast images (*upper*) and SEM analysis (*lower*) of SS-2 cells cultured with or without under 10 ng/mL TGF-β1 for 2. Original magnification, ×100; inset, ×200; scale bars 10 μm. (B) qRT-PCR of EMT markers in SS-2 cells cultured with or without 10 ng/mL TGF-β1 for 2 d. Results are presented as the mean ± SD of three independent experiments; *p < 0.05, **p < 0.01. (C) Immunocytochemical staining in SS-2 cells cultured with or without 10 ng/mL TGF-β1 for 2 d. Representative images are shown (E-cadherin, *red*; DAPI, *blue*). scale bars 20 μm. (D) Matrigel invasion assays performed in SS-2 cells cultured with or without 10 ng/mL TGF-β1 for 2 d. Representative results from measurements of 12 fields are shown. **p < 0.01. EMT: epithelial–mesenchymal transition; NEC: neuroendocrine carcinoma; qRT-PCR: quantitative reverse transcription-polymerase chain reaction*;* SEM: Scanning electron microscopic*;* TGF- β: transforming growth factor-beta. (For interpretation of the references to color in this figure legend, the reader is referred to the Web version of this article.)

Next, we examined whether EMT could be induced in SS-2 cells by TGF-β1 treatment. Non-treated SS-2 cells exhibited dome-like morphology by phase contrast and SEM analysis, whereas TGF-β1-treated SS-2 cells showed spindle-like morphology (Fig. 2A). We further examined the expression levels of the EMT markers. Real-time qPCR analysis showed a significant reduction in epithelial markers (*E-cadherin*) and increase in mesenchymal markers (*N-cadherin*, *Slug*, and *Vimentin)* in TGF-β1-treated SS-2 cells (Fig. 2B). In contrast, the expression of *Snail*, a transcriptional repressor of E-cadherin, was significantly decreased in TGF-β1-treated SS-2 cells. Immunocytostaining showed that E-cadherin protein was expressed at the cell-cell junctions of TGF-β1-treated SS-2 cells comparable to that in non-treated cells (Fig. 2C). These results suggested that TGF-β1-treated SS-2 cells were in a partial EMT state, which is an intermediate state between the epithelial and mesenchymal phenotypes. The motility of EMT-induced cells is known to be aggressive [18,19]. Invasion assay using Matrigel invasion chambers showed that the number of invading cells was significantly higher in TGF-β1-treated SS-2 cells than that in non-treated cells (Fig. 2D). These results indicated that TGF-β1 stimulation induced partial EMT in the NEC cell line SS-2, in which the expression of E-cadherin was maintained, leading to enhanced invasiveness. Partial EMT has been implicated in the collective migration of cancer cells [20,21]. Even after plating of single dissociated TGF-β1-treated SS-2 cells, almost all these cells seemed to form aggregated colonies on the invasion chamber (not shown), reflecting the collective invasion in TGF-β1-treated SS-2 cells. The enhanced stemness and/or drug resistance traits of partial EMT cells as compared to fully epithelial or fully mesenchymal cells have been reported in several types of cancer [[22], [23], [24], [25], [26]]. Therefore, induction of partial EMT in the NEC cell line SS-2 may be associated with greater metastatic potential and worse prognosis in colorectal NEC.

### 3.3 Extracellular membrane degradation activity and adhesion ability in NEC cell line SS-2

**Fig. 3:**
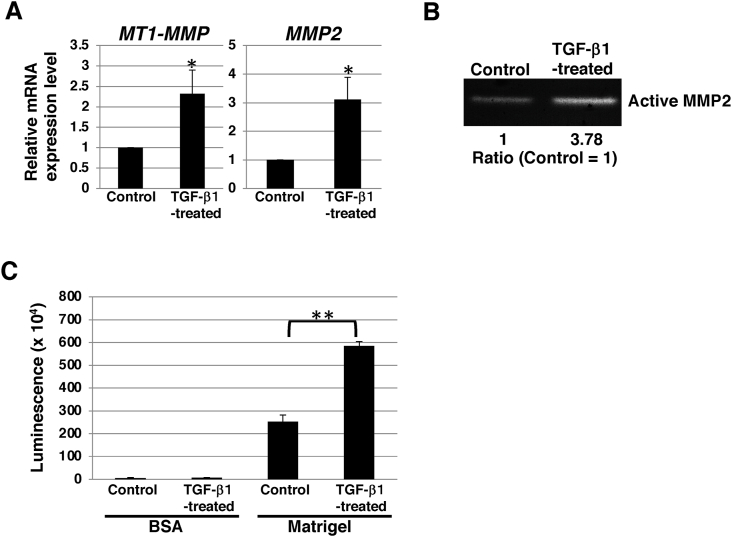
**ECM degradation activity and adhesion ability in NEC cell line SS-2.** (A) qRT-PCR of *MT1-MMP* and *MMP2* in SS-2 cells cultured with or without 10 ng/mL TGF-β1 for 2 d. Results are presented as the mean ± SD of three independent experiments; *p < 0.05. (B) Gelatin zymography was performed using culture supernatants from SS-2 cells that were cultured with or without 10 ng/mL TGF-β1 for 2 d. Relative band intensity is shown. (C) Adhesion assays in SS-2 cells cultured with or without 10 ng/mL TGF-β1 for 2 d. Each cell type exhibited negligible adhesion to BSA. **P < 0.01. ECM: extracellular membrane; MMP: matrix metalloproteinase; NEC: neuroendocrine carcinoma; qRT-PCR: quantitative reverse transcription-polymerase chain reaction*;* TGF- β: transforming growth factor-beta.

Increased production of MMPs is a key feature of invasion [27]. Next, we examined the expression levels of MMP and its activity in TGF-β1-treated SS-2 cells. Real-time qPCR analysis showed that *MT1-MMP* and *MMP2* mRNA levels were upregulated in TGF-β1-treated SS-2 cells than in non-treated cells (Fig. 3A). Activation of MMP2 is known to be induced by MT1-MMP by proteolytic cleavage of the prodomain of proMMP2 [28,29]. Next, we examined the activity of MMP2 released from TGF-β1-treated SS-2 cells using gelatin zymography. As shown in Fig. 3B, MMP2 activity was higher in TGF-β1-treated SS-2 cells than in non-treated cells. During invasion, a critical event is the adhesion of cancer cells to the extracellular matrix [30]. We then investigated the cell-to-matrix adhesion. Adhesion assays on Matrigel revealed that TGF-β1-treated SS-2 cells exhibited a statistically significant increase in adhesiveness (Fig. 3C). These results indicated that increased MMP activity and adhesion ability contributed to the invasiveness of the NEC cell line SS-2 after EMT induction.

### 3.4 α2-integrin dependent adhesion and invasion in NEC cell line SS-2

**Fig. 4:**
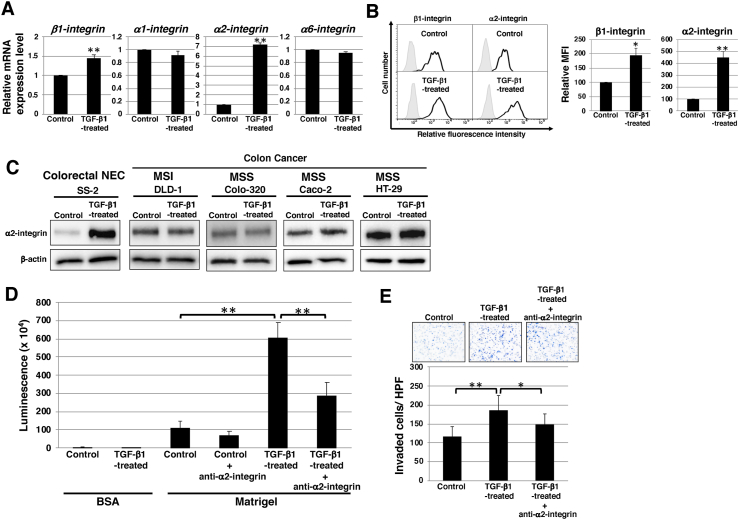
**α2-integrin dependent adhesion and invasion in NEC cell line SS-2.** (A) qRT-PCR of *integrins* in SS-2 cells cultured with or without 10 ng/mL TGF-β1 for 2 d. Results are presented as the mean ± SD of three independent experiments; **P < 0.01. (B) FACS of β1-integrin and α2-integrin levels in SS-2 cells cultured with or without 10 ng/mL TGF-β1 for 2 d. Controls are indicated by thin lines with gray color. MFIs relative to non-treated control cells are shown on the right side. Results are presented as means ± SD from three independent experiments. *P < 0.05, **P < 0.01. (C) Immunoblotting for α2-integrin and β-actin (loading control) in SS-2 and colon cancer cells cultured with or without 10 ng/mL TGF-β1 for 2 d. (D) Adhesion assays in SS-2 cells cultured with or without 10 ng/mL TGF-β1 for 2 d. Cells were treated with or without anti-α2-integrin antibody before assays. **P < 0.01. (E) Matrigel invasion assays performed in SS-2 cells cultured with or without 10 ng/mL TGF-β1 for 2 d. Cells were treated with or without anti-α2-integrin antibody before assays. Representative results from measurements of 12 fields are shown. **p < 0.01. FACS: fluorescence activated cell sorter; MFI: mean fluorescence intensity; NEC: neuroendocrine carcinoma; qRT-PCR: quantitative reverse transcription-polymerase chain reaction*;* TGF- β: transforming growth factor-beta.

Integrins, which mediate the crosstalk between tumor cells and basement membrane proteins, are major cell-surface receptors that play a critical role in cell adhesion, invasion, and metastasis induction [[31], [32], [33]]. Therefore, we investigated the expression levels of these integrins in TGF-β1-treated SS-2 cells. Real-time qPCR analysis showed that *β1-integrin* and *α2-integrin* mRNA levels were significantly upregulated in TGF-β1-treated SS-2 cells (Fig. 4A). Furthermore, FACS analysis confirmed that the cell surface levels of β1-integrin and α2-integrin were markedly increased in TGF-β1-treated SS-2 cells (Fig. 4B). Western blot analysis showed upregulation of α2-integrin expression after TGF-β1 treatment in the NEC cell line SS-2, but not in other colon cancer cell lines, DLD-1, Colo-320, Caco-2, and HT-29 (Fig. 4C). This result suggested that upregulation of α2-integrin expression during EMT induction was a unique feature of NEC cells. We further examined the dependency of α2-integrin on cell-to-matrix adhesion in TGF-β1-treated SS-2 cells. Adhesion assays revealed that the anti-α2-integrin blocking antibody inhibited the increased adhesion on Matrigel in TGF-β1-treated SS-2 cells (Fig. 4D), indicating that the enhanced adhesiveness of these cells was dependent on α2-integrin. Finally, we examined whether increased adhesion mediated by α2-integrin led to the invasiveness of the NEC cell line SS-2 after EMT induction. The increased invasion of TGF-β1-treated SS-2 cells was inhibited by the anti-α2-integrin blocking antibody (Fig. 4E). These results indicated that upregulation of α2-integrin during EMT induction contributed to the invasiveness of the NEC cell line SS-2.

In conclusion, we have demonstrated that TGF-β1 stimulation induces partial EMT and maintains E-cadherin in the NEC cell line SS-2, leading to enhanced invasiveness dependent on upregulation of α2-integrin. Therefore, α2-integrin expression in NEC cells during EMT may be correlated with a malignancy potential. These results suggest that targeting α2-integrin may represent a novel therapeutic option for metastasis of colorectal NEC cells; however, further studies are required to clarify the involvement of α2-integrin in the metastasis of colorectal NEC cells *in vivo*.

## Author contributions

N.S., S.S., and T.I. designed the research and wrote the paper. Y.S. performed the electron microscopy experiments. T.A. and H.Y. reviewed and edited the document and provided helpful discussions. T.Y., G.T., H.S., A.M., T.I., K.T., K.Y., K.U., S.K. and T.M. reviewed and edited the content of the manuscript. All authors have read and agreed to the published version of the manuscript.

## Declaration of interests

The authors declare that they have no known competing financial interests or personal relationships that could have appeared to influence the work reported in this paper.
